# Periodic stub implementation with plasmonic waveguide as a slow-wave coupled cavity for optical refractive index sensing

**DOI:** 10.1038/s41598-024-55618-0

**Published:** 2024-03-02

**Authors:** Zahra Sadat Tabatabaeian, Fatemeh Kazemi, Ferdows B. Zarrabi

**Affiliations:** 1 Department of Electrical Engineering, Faculty of Engineering, University of Guilan, Rasht, Iran; 2 Faculty of Engineering, University of Zabol, Zabol, Iran; 3 Department of Engineering, Science and Research Branch, Islamic Azad University, Tehran, Iran

**Keywords:** Microfluidic, Refractive index sensing, Dual-band, Coupled cavity, Coupled mode theory, Electrical and electronic engineering, Optical materials and structures, Optical physics, Nanoscale materials

## Abstract

Optical biosensors based on plasmonic nanostructures have attracted great interest due to their ability to detect small refractive index changes with high sensitivity. In this work, a novel plasmonic coupled cavity waveguide is proposed for refractive index sensing applications. The structure consists of a metal–insulator–metal waveguide side coupled to an array of asymmetric H-shape element, designed to provide dual-band resonances. The sharp transmission dips and large field enhancements associated with dual-band resonances can enable sensitive detection of material under test. The resonator array creates a slow light effect to improve light-matter interactions. The structure was simulated using the finite integration technique as the full-wave technique, and the sensitivity and figure of merit were extracted for different ambient refractive indices. The maximum sensitivity of 1774 nm/RIU and high figure of merit of 2 × 10^4^ RIU^−1^ for the basic model and 1.15 × 10^5^ RIU^−1^ for the modified model were achieved, demonstrating the potential for high-performance sensing. The unique transmission characteristics also allow for combined spectral shaping and detection over a broad bandwidth. The simple, compact geometry makes the design suitable for on-chip integration. This work demonstrates a promising refractive index sensor based on coupled dual-band resonators in a plasmonic waveguide.

## Introduction

Plasmonics is known as a unique optical property of metals, specifically the coupling of light with free electron oscillations known as surface plasmon polaritons (SPPs)^1^. This field has enabled the development of novel photonic devices at the nanoscale. Exciting applications include plasmonic waveguides for confining and routing light below the diffraction limit^2^, ultra-thin optical absorbers^3^, nanoantennas for enhancing light emission and detection^4^, optical switches controlled by external stimuli^3^, and logic gates for all-optical computing^5^. Many plasmonic structures can function as optical sensors by monitoring changes in the local refractive index, based on the high sensitivity of SPP resonances^6^. Binding of liquids or a change in the surrounding environment induces a spectral shift in the plasmon response^7^, which can be accurately measured using extinction or reflected light spectroscopy. The magnitude of the wavelength shift per refractive index unit change provides the sensitivity. This can be optimized along with the figure of merit, which accounts for resonance sharpness^8^, by engineering the nanostructure geometry and materials.

Metal–insulator–metal (MIM) and metal–dielectric-metal (MDM) waveguide structures have been found widespread use for nanophotonic applications^9,10^. These configurations allow confinement and propagation of electromagnetic waves within the dielectric or insulating layer sandwiched between metal films. They can be viewed as nanoscale transmission lines at optical frequencies^11^. The main advantageous of this object concentrate the optical energy within the small interior region between the metal layers. This field enhancement effect has been leveraged in plasmonic perfect absorbers, where the increased intensity amplifies light-matter interactions with the absorbing medium^12^. For sensing, the field concentration translates to larger wavelength shifts and increased sensitivity when liquid bind within this interior region. This also improves the figure of merit by sharpening the plasmon resonance peak^13^.

Recently, the MIM coupled waveguide has been used in various forms as optical sensor. The basic models have simple form with single stub^14,15^, multiple stubs for Fano response^16^ and the grating structures as a multi-band filter^17^. For obtaining sharp Fano response more complicated models have been suggested including metal–insulator–metal waveguide coupled with a disk and a ring cavity^18^, stub and groove resonator coupled^19^, multiple-ring shaped^15^, the circular cavity optimized by a metallic nanodisk^20^, dual H-Shaped cavities side-coupled waveguide^21^. Moreover, the filled cavities with other materials such as DNA^22^ and Kerr^23^ as reconfigurable structure or filled with silicon for increasing coupling have been presented^24^. The metamaterial structures has been considered for their special characteristic for designing microwave and optical devises and the split ring resonator (SRR) has been developed in verity of models where the gaps plays the role of capacitance and the metal strips are inductance^25^.

In this work, we have combined the concepts of spoof plasmonic structures as periodic elements and stub resonators in cavity waveguides to realize a novel geometry with enhanced bandwidth, Q-factor, and figure of merit. The proposed structure integrates a plasmonic waveguide exhibiting dual-band resonances with a spoof plasmonic grating to improve light-matter interactions. This leads to sharper dual-band resonances while expanding the operational bandwidth, enabling sensitive refractive index detection. The underlying principles and theoretical formulations for plasmonic waveguide design and stub resonators are outlined to provide context. The proposed integrated plasmonic cavity-stub structure is then presented along with potential fabrication methods using standard nanofabrication techniques. Geometric considerations for the waveguide, stub, and grating dimensions are detailed. The results showcase the transmission and phase response for three cases with 1, 3, and 5 stubs. Improved resonance depth and flattened phase profile is demonstrated as stub number increases due to the slow light effect. The coupled mode theory is fitted to the transmission spectra and parametric circuit modeling provides physical insights. Finally, the sensing application is evaluated by testing refractive index detection sensitivity.

## The theory of the optical waveguide

Consider an MDM waveguide. This waveguide consists of two metal layers which are considered infinite and a dielectric layer is placed between them. In this article, the metal layers are made of silver and the dielectric layers are considered to be made of air. If the thickness of the dielectric layer is less than the dielectric skin depth, the refractive index of the MDM waveguide is obtained from Eq. (1)^22^.

1 $$n_{eff} = \beta /k_{0}$$

In this equation, k_0_ = 2π/*λ*, *λ* = 2πc/ω which c is the speed of wave propagation in vacuum and *β* is obtained by solving Eq. (2)^22^.

2 $$\begin{gathered} \tanh \left( {\frac{{ik_{1} h}}{2}} \right) = \frac{{\varepsilon_{1} k_{2} }}{{\varepsilon_{2} k_{1} }} \hfill \\ k_{j} = \left( {\varepsilon_{j} k_{0}^{2} - \beta^{2} } \right)^{1/2} \hfill \\ \end{gathered}$$

In this equation, h is the thickness of the air region, ε_1_ is the relative permittivity of vacuum, and ε_2_ is the relative permittivity of silver. Equation 3 obtained from Drude–Lorentz model is used to calculate the permittivity of silver^26^.

3 $$\varepsilon_{2} \left( \omega \right) = 1 - \frac{{\omega_{p}^{2} }}{{\omega \left( {\omega + i\gamma } \right)}}$$

where ω_p_ = 2002.6 THz is the bulk plasma frequency of silver and γ = 11.61 THz is a damping constant. After calculating the refractive index from Eqs. (1), and (4) can be used to obtain the guided wavelength λ_MDM_ and propagation length L_SPP_ by using its real and imaginary parts, respectively^27^.

4 $$n_{eff} = \frac{\lambda }{{\lambda_{MDM} }} + i\frac{\lambda }{{4\pi L_{SPP} }}$$

**Figure 1:**
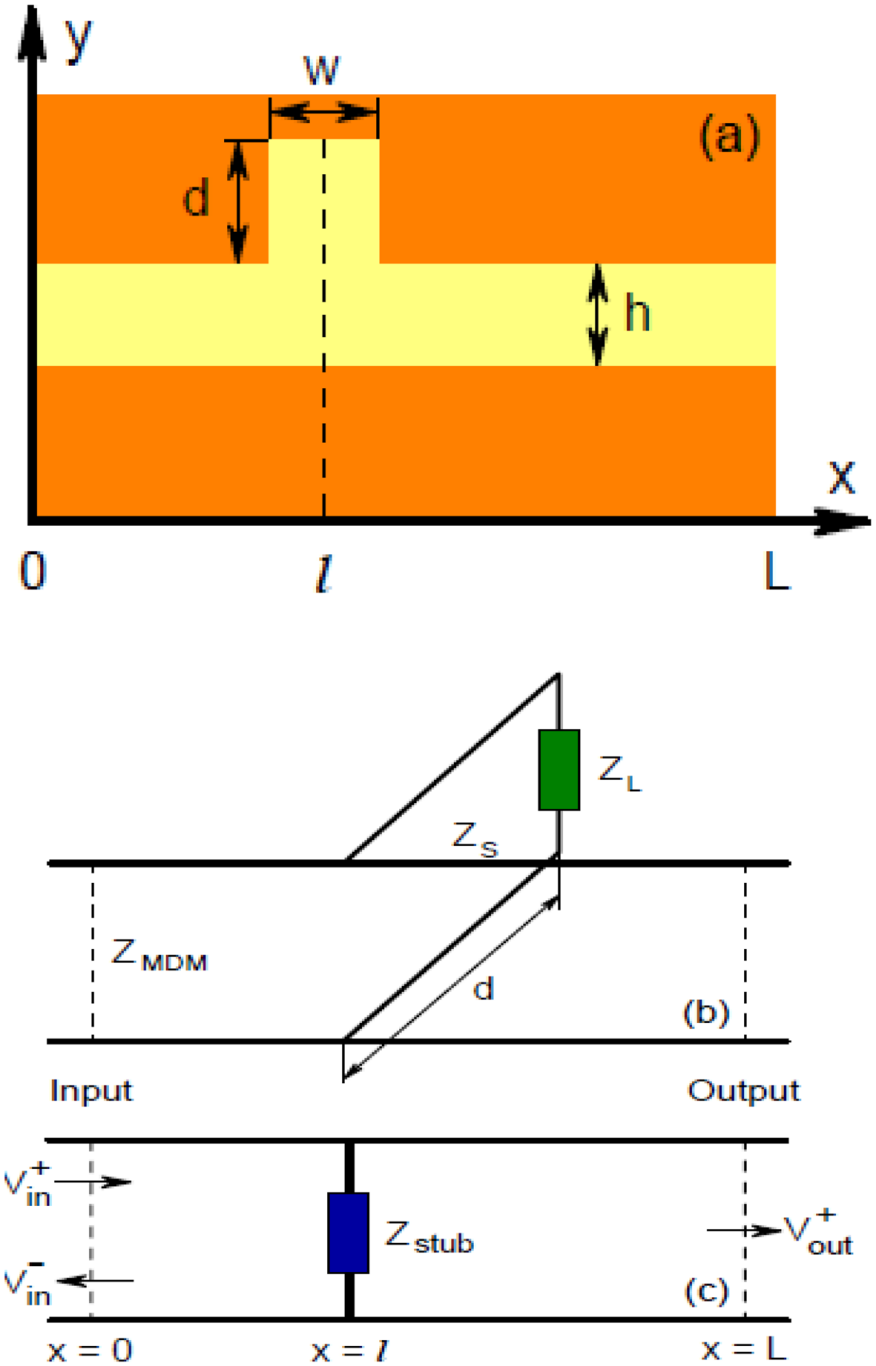
(**a**) MDM waveguide with a stub (**b**) The equivalent transmission line (**c**) Reduced equivalent transmission line^28^.

After obtaining the propagation characteristics of the MDM waveguide, suppose that a stub is placed in the path of the waveguide as shown in Fig. 1a. To obtain the transmission of this waveguide, it can be modeled with a transmission line. The equivalent transmission line of this structure is shown in Fig. 1b.

In this model, Z_S_ and Z_MDM_ are the characteristic impedance of the stub and waveguide, respectively, and are obtained from Eq. (5)^28^.

5 $$\begin{gathered} Z_{MDM} \simeq \frac{\beta \left( h \right)h}{{\omega \varepsilon_{0} \varepsilon_{1} }} \hfill \\ Z_{S} \simeq \frac{\beta \left( w \right)w}{{\omega \varepsilon_{0} \varepsilon_{1} }} \hfill \\ \end{gathered}$$

In this equation, ε_0_ is permittivity of vacuum, h is the thickness of the waveguide and w is the thickness of the stub, and *ꞵ*(*h*) and *ꞵ*(*w*) are obtained using Eq. (2) for the waveguide and stub, respectively^28^.

Now, to find the value of Z_L_ in the transmission line model, the absolute value of the reflection from the end of the stub in the two transmission line and waveguide models should be equal to each other^29^.

6 $$\begin{gathered} \Gamma = \frac{{Z_{L} - Z_{S} }}{{Z_{L} + Z_{S} }} = \frac{{\sqrt {\varepsilon_{2} } - \sqrt {\varepsilon_{1} } }}{{\sqrt {\varepsilon_{2} } + \sqrt {\varepsilon_{1} } }} \hfill \\ Z_{L} = \sqrt {\frac{{\varepsilon_{2} }}{{\varepsilon_{1} }}} Z_{S} \hfill \\ \end{gathered}$$

Therefore, the input impedance of the stub is obtained by applying the transmission line theory from Eq. (7)^28^.

7 $$Z_{stub} = \frac{{Z_{L} - iZ_{S} \tan \left( {\beta \left( w \right)d} \right)}}{{Z_{S} - iZ_{L} \tan \left( {\beta \left( w \right)d} \right)}}$$

where *d* is the length of the stub.

Having a transmission line model, the transmission matrix is obtained from Eq. (8) and transmission is obtained from the transmission matrix as shown in Eq. (9)^29^.

8 $$\begin{gathered} T = A\left( l \right)BA\left( {L - l} \right) \hfill \\ A\left( x \right) = \left[ {\begin{array}{*{20}c} {e^{ - i\beta \left( h \right)x} } & 0 \\ 0 & {e^{i\beta \left( h \right)x} } \\ \end{array} } \right],B = \left[ {\begin{array}{*{20}c} {1 + \frac{{Z_{MDM} }}{{2Z_{stub} }}} & {\frac{{Z_{MDM} }}{{2Z_{stub} }}} \\ { - \frac{{Z_{MDM} }}{{2Z_{stub} }}} & {1 - \frac{{Z_{MDM} }}{{2Z_{stub} }}} \\ \end{array} } \right] \hfill \\ \end{gathered}$$

where *l* is the location of the stub and *L* is the length of the waveguide

9 $$T_{1} = \left| {\frac{{V_{out}^{ + } }}{{V_{in}^{ + } }}} \right|^{2} = \left| {1 + \frac{{Z_{MDM} }}{{2Z_{stub} }}} \right|^{ - 2} e^{{\left( { - \frac{L}{{L_{SPP} }}} \right)}}$$

By increasing the number of stubs, the transmission matrix is changed and the transmission is obtained from it.

## Design optical waveguide

**Figure 2:**
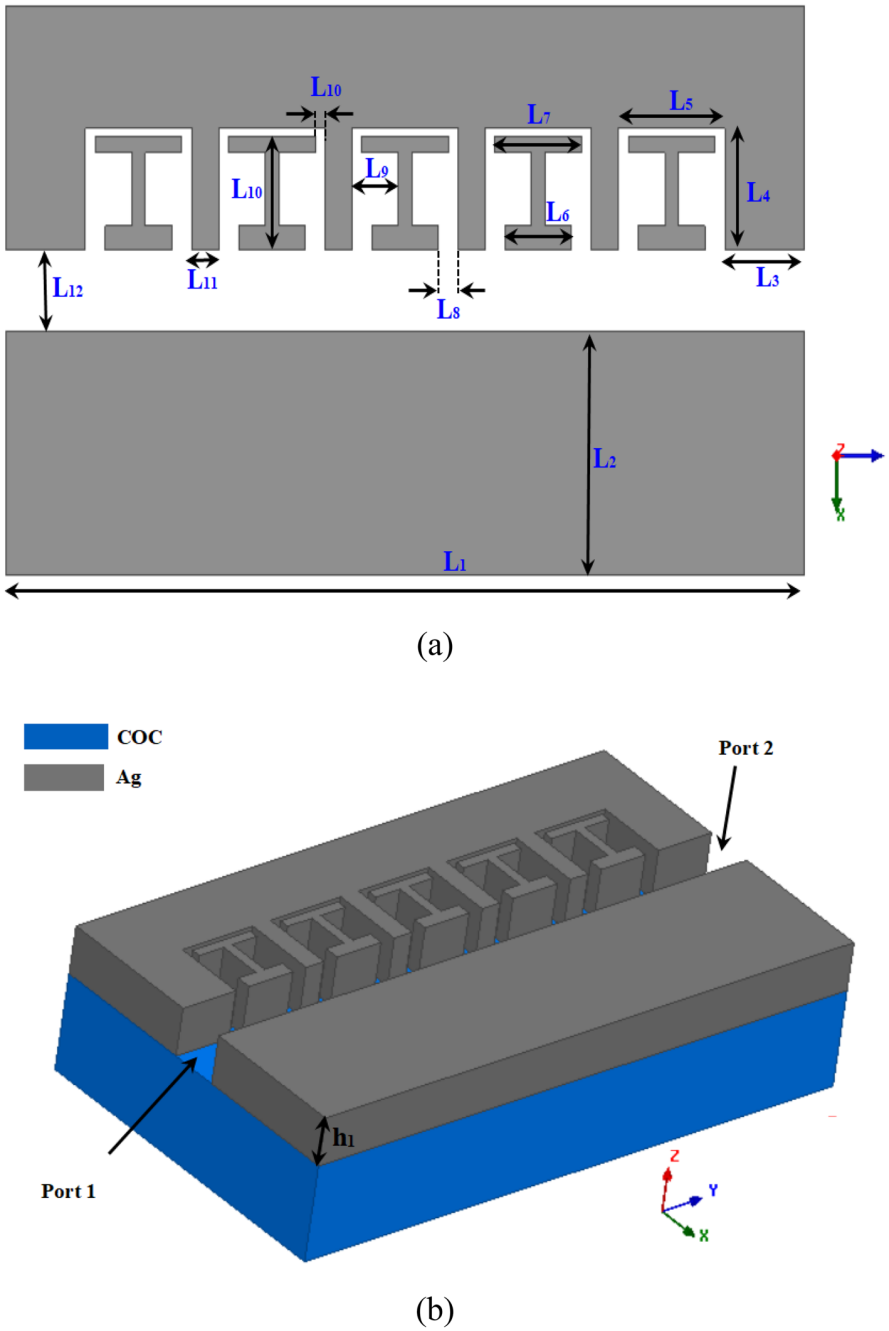
(**a**) The geometry of the proposed optical waveguide with cavities array (**b**) The 3D view of the proposed device over the substrate, all dimension of the waveguides for the basic model are L_1_ = 1200 nm, L_2_ = 300 nm, L_3_ = 120 nm, L_4_ = 150 nm, L_5_ = 160 nm, L_6_ = 100 nm, L_7_ = 130 nm, L_8_ = 30 nm, L_9_ = 70 nm, L_10_ = 140 nm, L_11_ = 40 nm, L_12_ = 100 nm.

Figure 2 shows the main structure of the proposed plasmonic waveguide cavity. As depicted, the structure consists of a silver plasmonic waveguide is placed on a transparent material such as COC (cyclic olefin copolymer) substrate^30^, which is nearly transparent at the operation frequencies. The Johnson model was used to model the silver. The proposed structure has a silver surface with width of 700 nm (2 × L_2_ + L_12_) and length of 1200 nm (L_1_). The height of the silver surface is assumed to be 100 nm (h_1_). Long slit is created at the center of the structure to form a plasmonic waveguide with A 100 nm (L_12_) wide and 1200 nm. A square slot with dimensions of 150 × 160 nm (L_4_ × L_5_) is designed at the edge of the central slit in one side of the waveguide as a cavity. Then, an asymmetric H-shaped element is placed inside the cavity. The periodic structure, consisting of 5 elements, is then created with a cell separation of 40 nm (L_11_). The periodic structure allows for coupling of the incident light into surface plasmon polaritons (SPPs) modes of the waveguide. The central slot supports propagation of SPPs which can couple to the cavity region. The H-shaped element provides asymmetry needed for non-degenerate cavity modes. Varying the geometry of the H-element and periodic structure enables tuning of the cavity resonance wavelengths. The square notch further perturbs the field distribution, influencing the coupling efficiency.

Figure 2b shows a 3D view of the structure, depicting the excitation location. In this work, the proposed cavity structure is excited using a continuous-wave laser tuned to the resonance wavelength. The laser beam can be used onto one side of the periodic structure. This allows efficient coupling of light into the SPP modes of the plasmonic waveguide. The structure is simulated using CST Microwave Studio, implementing the finite integration technique (FIT) numerical method. The plasmonic waveguides support TE mode propagation and appropriate boundary conditions were applied to excite this mode. Two waveguide ports were utilized at the input to excite the structure. A hexahedral legacy mesh is implemented to discretize the geometry. This provides sufficient resolution to accurately model the plasmonic and cavity effects.

Although this work is a simulation base study, the conventional process of fabrication can be suggested as a routine method and the plasmonic waveguides and cavity structures can be fabricated using conventional nanofabrication techniques that are widely used for photonic devices.

This typically starts with a substrate such as silicon, glass or COC as a transparent material which is coated with a thin film of the plasmonic metal, commonly silver or gold. The metal film can be deposited using physical vapor deposition methods such as thermal evaporation or sputtering. The thickness is chosen based on the target operating wavelength, with 100 nm being typical^31,32^.

The waveguide and cavity layout is then patterned using electron beam lithography (EBL). This involves spinning a thin resist layer on the metal surface and selectively exposing the desired pattern by using a focused electron beam. The exposed resist is dissolved and the pattern is transferred to the metal film using ion milling or wet chemical etching^31,32^.

The remaining resist can be stripped to realize the final metallic nanostructures. EBL provides the resolution and alignment accuracy needed for the sub-wavelength features. The final step is deposition of the dielectric layer that surrounds the metallic elements. This low-index material acts as optical cladding for the plasmonic waveguides. Various materials can be used such as polymers, oxides, or fluorides. The dielectric thickness and properties impact the waveguide performance. The dielectric deposition can utilize techniques such as chemical vapor deposition, atomic layer deposition, or spin coating depending on the desired material. This completes the basic fabrication process flow for realizing the simulated plasmonic cavity designs^31,32^.

## Simulation and experimental result and discussions

The periodic elements scatter the propagating SPPs, enabling a portion of the energy to couple into the cavity region through the square notch. At resonance, the cavity builds up large field intensity enhancements inside the gap. The enhanced optical fields can be used for surface-enhanced Raman spectroscopy and other applications requiring strong light-matter interactions. Varying the excitation wavelength around the cavity resonance enables tuning of the field enhancement.

**Figure 3:**
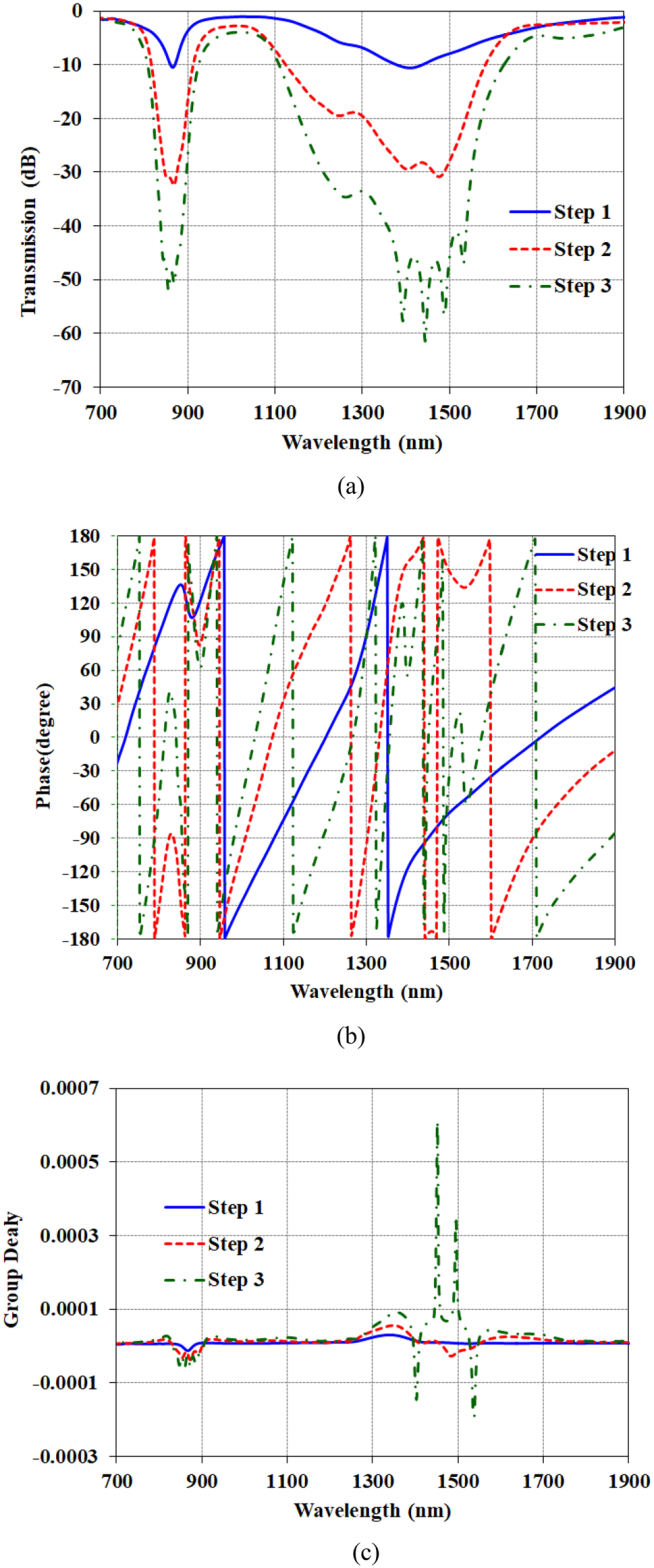
The comparison of the transmission and phase of three steps of the design with Stub 1, Stub 3 and Stub 5 (**a**) The transmission of the waveguide (**b**) The phase of the waveguide (**c**) The group delay of the waveguide.

The transmission characteristics of the proposed coupled cavity structure were analyzed for different configurations of the cavity array, as shown in Fig. 3a. The structure consists of periodic cavities designed to provide a slow-wave effect, as depicted in Fig. 1. Therefore, the transmission parameter S_21_ is analyzed for three different cases of step 1—with only 1 cavity, step 2—with 3 cavities and step 3—with 5 cavities.

First for the step 1, the transmission is simulated with only a single cavity element. The results in Fig. 2a show two resonance dips appearing around 900 nm and 1350 nm wavelengths, with transmission dropping to approximately − 10 dB at these points.

Next for the step 2, the number of cavities is increased to three cavities. In this case, the resonance dips became much sharper, with the transmission reducing to − 30 dB for both resonances across the same spectral range. Finally for the step 3, for an array with five cavity elements, the transmission reduction significantly improved further. The first resonance reached − 50 dB while the second dipped down to -62 dB transmission.

Importantly, as the number of coupled cavities increased, the overall bandwidth of the structure exhibited substantial broadening. This enables two potential applications—refractive index sensing using the sharp resonant dips, as well as optical filtering over the wide bandwidth. By tuning the cavity and array design, the proposed structure could provide combined spectral shaping and detection capabilities on a nanophotonic chip. Further optimizations may maximize performance for the desired functionality.

Figure 3b shows the phase of S_21_ (transmission) response for the three cases of one stub, three stubs, and five stubs. It can be observed that the spoof plasmonic structure primarily controls the phase. As the number of stubs increases, the phase variations become more constrained over a wider wavelength range around the resonance peaks. For the single stub case, abrupt phase changes are seen at the resonant wavelengths of 900 nm and 1350 nm. When three stubs are introduced, the phase variations smooth out somewhat but there are still relatively steep phase slopes around the resonances. However, with five coupled stubs, the transmission phase exhibits only gradual changes over a broad spectral width spanning the two resonances.

The flattened, fast-varying phase response arises due to the coupled cavities providing a slow light effect. As light propagates down the periodic stub array, its group velocity decreases significantly. This enhances the light-matter interaction length and builds up the field intensity in each cavity. Consequently, the broader phase profile is retained over an expanded wavelength range. The flattened phase leads to increased bandwidth while maintaining high quality factor resonances. Engineering the cavity spacing and dimensions provides control over the dispersion properties to achieve the desired phase and spectral response. Overall, the analysis indicates the significance of the spoof plasmonic geometry in shaping the transmission phase for broadband filtering applications. Further design optimization can balance the phase profile and resonance characteristics for both spectroscopy and wavelength selectivity functions. We generally have two concepts about bandwidth. First, there is the total width, which we often measure with a value of − 10 dB, and the second concept, which is actually − 3 dB, where we discuss the peaks. Here, using the slow wave structure is considered to enhance both concepts.

The group velocity of the transmission lines will decrease by the implementation of the slow wave structure, according to these attributes the group delay will increase in comparison with the conventional transmission line as the fast-wave structures. In Fig. 3c, the comparison between the group delays of the three steps is presented and the results prove that by increasing the number of the stubs, the slow wave characteristic is enhanced and the group delay of the structure is increased from 9.52295E−06 for 1 stubs structure to 6.1E−04 for 5 stubs structure.

Due to the form of sensor transmission, we are facing EIT phenomenon. Electromagnetically induced transparency (EIT) is a fascinating phenomenon that allows a typically opaque medium to become transparent to a specific range of light frequencies. This effect is achieved by utilizing two laser beams with varying frequencies to excite a group of atoms or molecules within the medium. The presence of a strong control field causes a shift in the energy levels of the atoms, which results in the absorption of one laser beam being negated by the emission of the other. This cancellation creates a transparent window in the frequency spectrum of the medium. EIT has numerous practical applications in quantum information processing, optical communications, and precision measurement^33^.

Therefore, the sensor can be modeled with Oscillators Model. The Oscillator Model is a fundamental physics model that explains the behavior of two-pole systems. The system is divided into two parts, each comprising of a pole and an oscillator, where the poles are connected to the oscillators. The model is represented by a set of coupled differential equations with two variables. Specifically, the interaction between the bright (*x*_*1*_) and quasi-dark (*x*_*2*_) oscillators with an incoming electric field E_0_e^iω^ can be quantitatively described using these differential equations by Eq. (10) and (11)^34^.:

10 $$\ddot{x}_{1} (t) + \gamma_{1} \dot{x}_{1} (t) + \omega_{0}^{2} x_{1} (t) + \Omega x_{2} (t) = gE$$

11 $$\ddot{x}_{2} (t) + \gamma_{2} \dot{x}_{2} (t) + (\omega_{0} + \delta )^{2} x_{2} (t) + \Omega x_{1} (t) = 0$$

where *γ*_*1*_* and γ*_*2*_ are damping of two oscillators, *x*_*1*_ and *x*_*2*_ the resonant amplitude of two oscillators, *ꞷ*_0_ is transparency frequency, *Ω* is the coupling between the two oscillators, *ẟ* represents the frequency difference between the intrinsic resonance frequency and transparency frequency, and g is the coupling strength between bright mode oscillator and the external field.

In the above two differential equations, if it is assumed that x_n_ = c_n_e^iꞷt^ (n = 1, 2) and the approximation of ꞷ_1_^2^-ꞷ^2^≈-2ꞷ_1_(ꞷ-ꞷ_1_) is used, the transmission is obtained in the form of Eq. (12)^34^:

12 $$T = 1 - {\text{Re}} \frac{{ig^{2} \left( {\omega - \omega_{0} - \delta + i\gamma_{2} /2} \right)}}{{\left( {\omega - \omega_{0} + i\gamma_{1} /2} \right)\left( {\omega - \omega_{0} - \delta + i\gamma_{2} /2} \right) - \Omega^{2} /4}}$$

**Figure 4:**
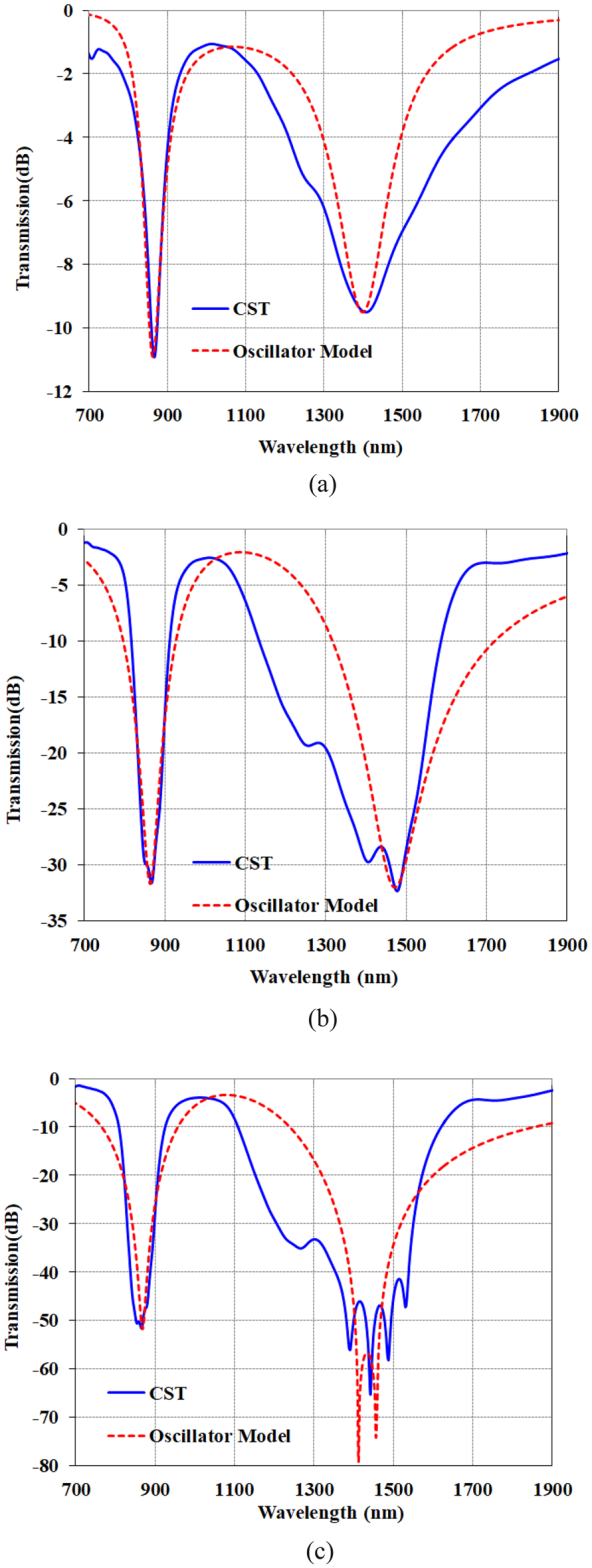
Comparison of transmission diagram obtained from CST software and oscillator model (**a**) 1 stub (**b**) 3 stubs (**c**) 5 stubs.

Here, for the three cases of one, three and five stabs, the transmission diagram is obtained through full wave analysis, with the help of CST software and the Oscillator Model, and compared with each other in Fig. 4. As can be seen, the Oscillator Model models this structure well.

The parameters of the Oscillator Model for three cases of one, three and five stabs are shown in Table 1.

**Table 1:** the parameters of the oscillator model for three cases of one, three and five stab.

	g	ꞷ_0_	ẟ	γ_1_	γ_2_	Ω
1stub	11.6	282.5 × 2π	− 18.2	50	357	870
3stubs	22.16	275.5 × 2π	1.2	820	200	870
5stubs	26.753	276.5 × 2π	10	1250	200	800

The lack of similar reproduction simulation results by CMT, which can be seen in Fig. 4, can be mainly attributed to two issues:

1. In general, the CMT method is for a narrow band and the structures whose resonances are very close to each other, so we can see in Fig. 4a that CMT has shown a more accurate model than the structure in Fig. 4b,c.
2. In the structure of 3 stubs and 5 stubs, internal couplings are formed that can create hybrid modes that cannot be analyzed, as a result, a wide band is created, but CMT can model based on the main modes.

As can be seen in Fig. 4, the CMT (coupled mode theory) method can justify the behavior of the desired sensor, but it is not possible to achieve the same results between the CMT method and the simulation techniques. The main reason for this dissimilarity is the coupling between elements and the parasitic capacitances in the cavity structure, which causes inaccurate modeling. As shown in Fig. 4a, the main capacitance for the first resonance at 866 nm is denoted as C_m1_. However, other parasitic capacitances of C_p1_ and C_p2_ are also present. Additionally, for the array element as shown in Fig. 4a, the coupled capacitance can be considered (C_couple_). C_m2_ is the main capacitance of the second resonance, but C_p4_ and C_p3_ can also be seen for this 1400 nm resonance. This simulation demonstrates coherence between the two resonances and the parametric studies in Figs. 7 and 8 confirm this. Therefore, in this case we do not have two separate resonators and CMT cannot fit the simulation.

**Figure 5:**
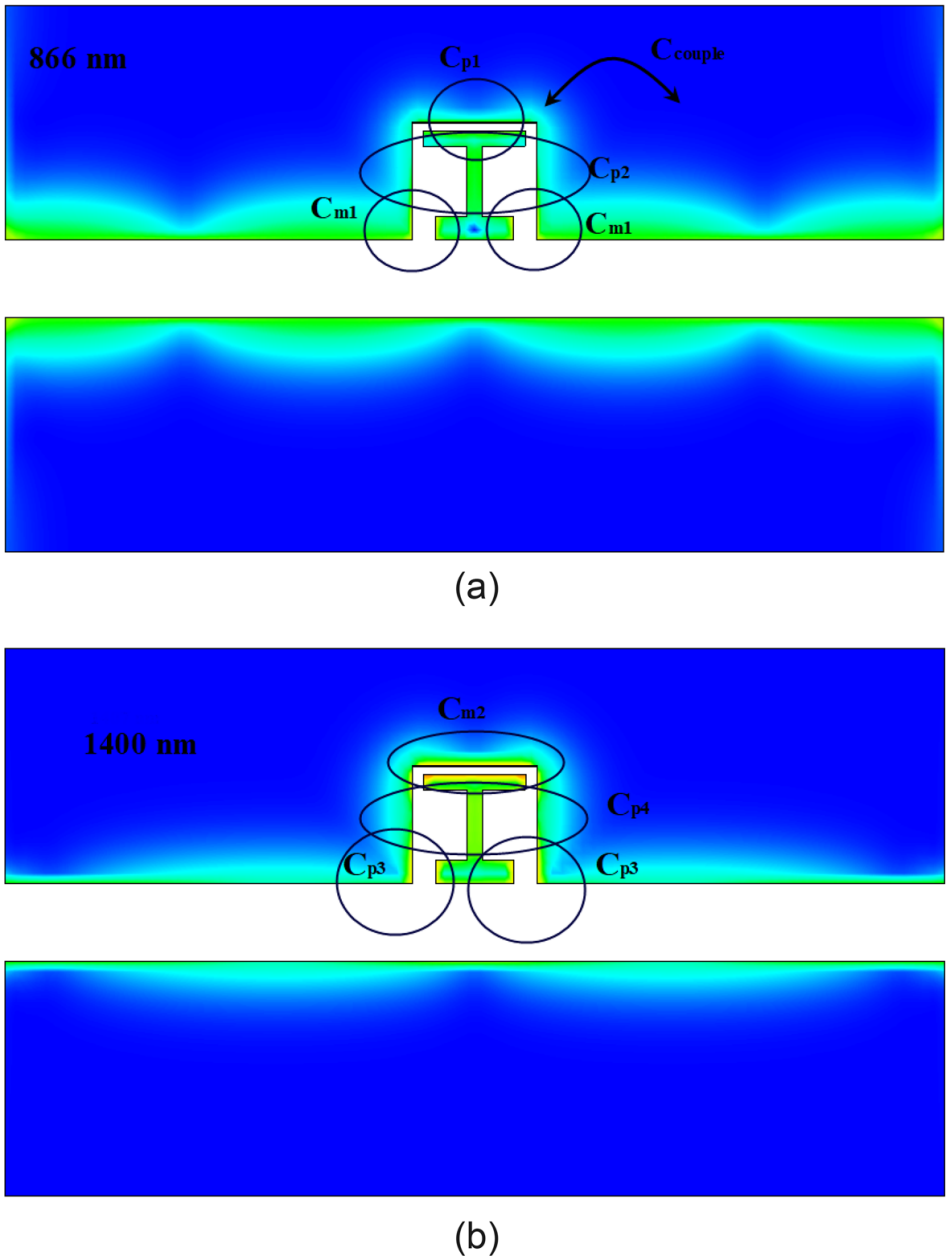
the electrical field and capacitances for the single stub element (**a**) for 866 nm (**b**) for 1400 nm.

**Figure 6:**
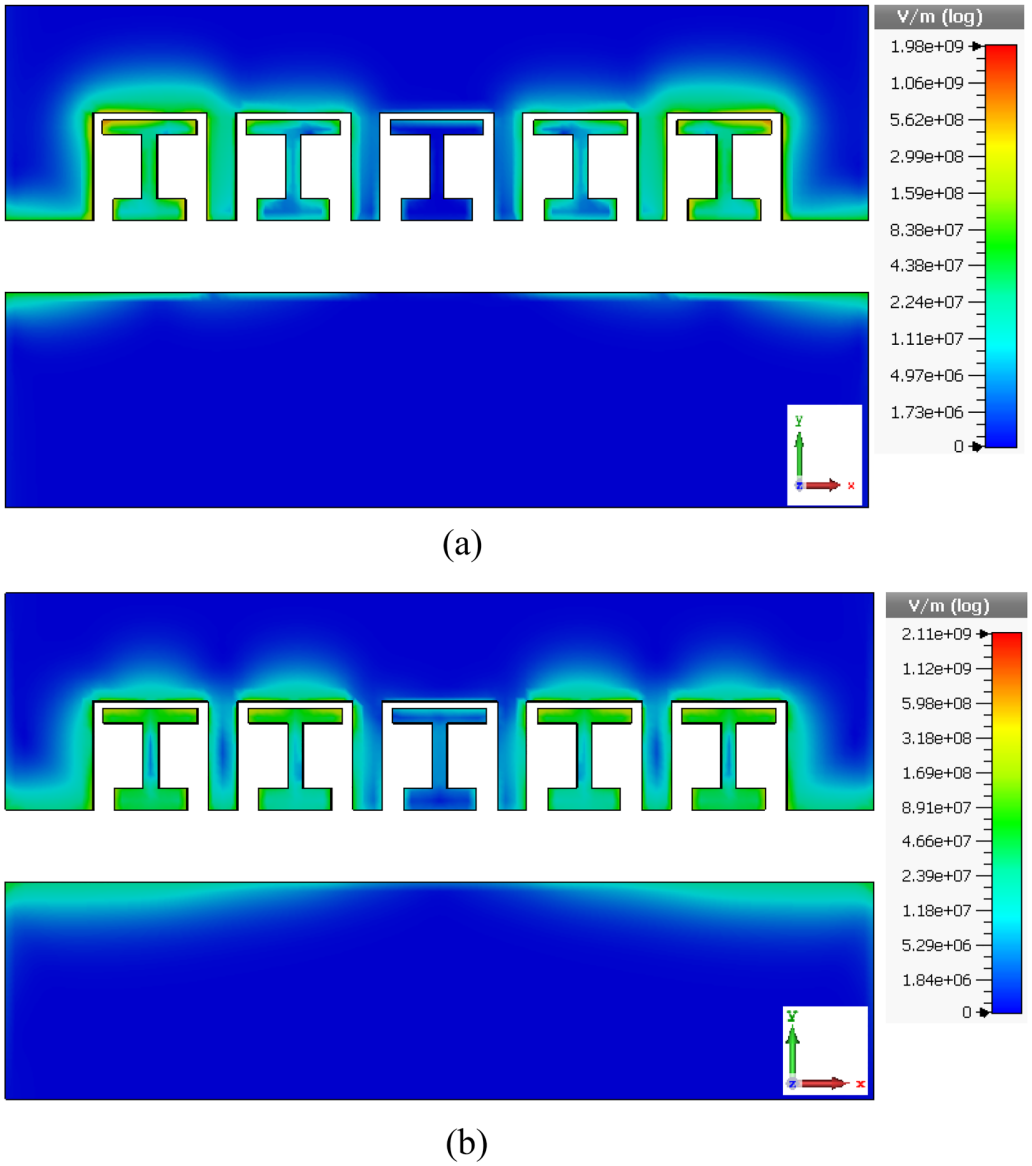
The electric field distribution of the cavity waveguide (**a**) at the first resonance at 210 THz (**b**) at the first resonance at 350 THz.

Moreover, these capacitors are shown in Fig. 5. On the other hand, in the field distribution in Fig. 6, we can see that the field distribution is non-uniform. This non-uniformity in practice causes the creation of different resonators and couplings between the cavities. As a result, while the structure transitions from a single-band state without the H element to dual-band with the H element, a high bandwidth is also achievable.

Figure 6 depicts the electric field distribution of the proposed plasmonic waveguide structure. It can be observed that the fields couple into the cavity regions. The coupling efficiency reduces for cavities positioned further from the input, as evidenced by the weaker fields. This explains the lower transmission for structures with fewer stub elements, resulting in a weaker filtering effect.

The changing field profile also indicates that the capacitive and inductive strengths vary along the waveguide length from the input to the center. This gives rise to the ripples in the spectral response of the filter. Figure 6a shows the field at 210 THz, where the first capacitor is formed between the H-shaped element and stub end. In Fig. 6b at 350 THz, the second capacitor is present between the H-element and start of the stubs.

Consequently, there are two capacitors and one inductor within the structure, with the capacitive coupling being frequency-dependent. This accounts for the two resonances with rippling observed in the transmission characteristic. By engineering the stub and cavity design, the capacitive and inductive effects can be tailored to achieve the desired spectral filtering response. The field distribution provides insights into optimizing the waveguide geometry for enhancing the capacitive coupling at targeted wavelengths.

In short, analysis of the plasmonic waveguide's electric field profile reveals the underlying capacitive and inductive effects that shape the filtering behavior. The design could be further refined by numerical optimization of the cavity and periodic stub structures to strengthen the field coupling at required frequencies. The proposed waveguide platform enables engineering the nanophotonic properties for sensing and spectral filtering functionalities.

Parametric analysis provides insights into the role of each element in shaping the equivalent circuit model, enabling optimization of the structure. Here, two key factors are studied. The first one is the length of the H- element.

**Figure 7:**
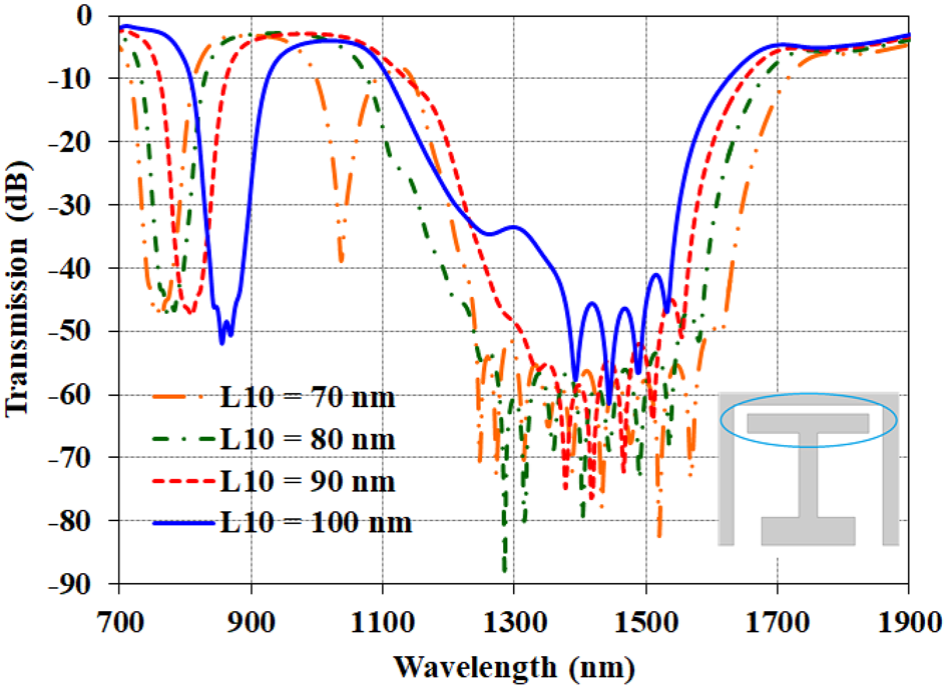
The transmission of the parametric studies for modifying the optical waveguide by changing the length of the H-element (L_10_).

Figure 7 shows the transmission response as the length of the H-shape element (L_10_) is varied. This metal section acts as the inductance can be denoted as L. Meanwhile, the gap size impacts the capacitance of C_2_. An illustration of the resonator is included for reference. Decreasing the L_10_ length is equivalent to increasing the gap at the stub end. However, the results indicate that shorter L_10_ redshifts the first resonance to lower wavelengths from λ_1_ = 850 nm for L_10_ = 100 nm to λ_1_ = 720 nm for L_10_ = 70 nm, meaning the inductance decreased based on $f_{1} = {1 \mathord{\left/ {\vphantom {1 {2\pi \sqrt {LC_{1} } }}} \right. \kern-0pt} {2\pi \sqrt {LC_{1} } }}$. As discussed in the electric field analysis, this capacitance (C_1_) arises from the H-shaped element at the start of the stub. At the second resonance, several effects are observed simultaneously. First, the frequency shifts due to variations in the L_10_ inductance length. Second, the bandwidth broadens, attributed to interactions between the two main capacitors. Third, the transmission depth decreases, likely from increased energy concentration in the created end-cavity region.

**Figure 8:**
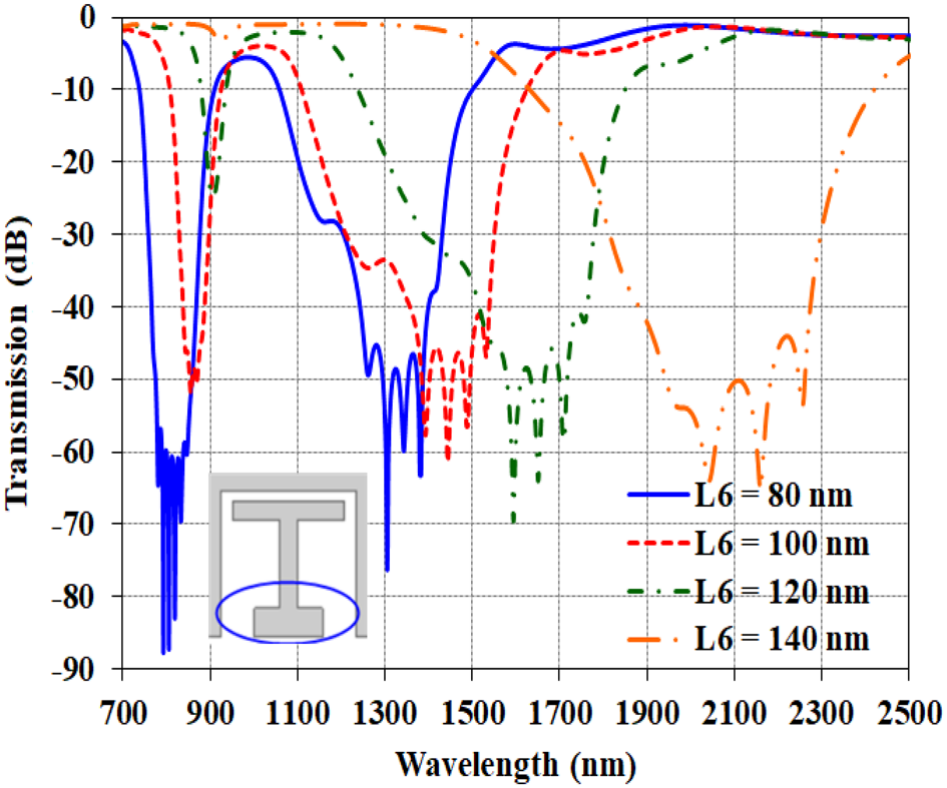
The transmission of the parametric studies for modifying the optical waveguide by changing the length of the downer branch of the H-element (L_6_).

As discussed, the H-shaped element acts as an inductor, while the two capacitors C_1_ and C_2_ create the dual resonances. The electric field analysis showed that at higher wavelengths, the capacitance at the start of the stubs plays the primary role as shown in Fig. 8. Examining the parametric results in Fig. 8, increasing the length of the lower H-shape branch L_6_ redshifts the second resonance to higher wavelengths for example form 1300 nm for L_6_ = 80 nm is shifted to 2100 nm for L_6_ = 140 nm. In other words, the frequency decreases, indicating an increase in the capacitance by $f_{1} = {1 \mathord{\left/ {\vphantom {1 {2\pi \sqrt {LC_{2} } }}} \right. \kern-0pt} {2\pi \sqrt {LC_{2} } }}$. Although some effects on the first resonance are seen, the second resonance is more strongly influenced by this capacitance variation. This aligns with the field distributions showing both capacitors impacting each resonance, but with greater individual dominance.

Secondly, reducing L_6_ to 80 nm brings the two resonances closer together compared to 140 nm. Meanwhile, the transmission depth reaches − 90 dB, signifying much higher quality factors. This is attributed to increased energy storage in the enhanced capacitances. Consequently, both the sensitivity and figure of merit improve. In short, sweeping the H-element dimensions provides levers to tune the capacitances responsible for each resonance. Careful engineering of these lengths can optimize the balance between resonance spacing, quality factor, and transmission depth. This parametric analysis forms a basis for designing the geometry to maximize sensing performance through independent control of the two resonances.

**Figure 9:**
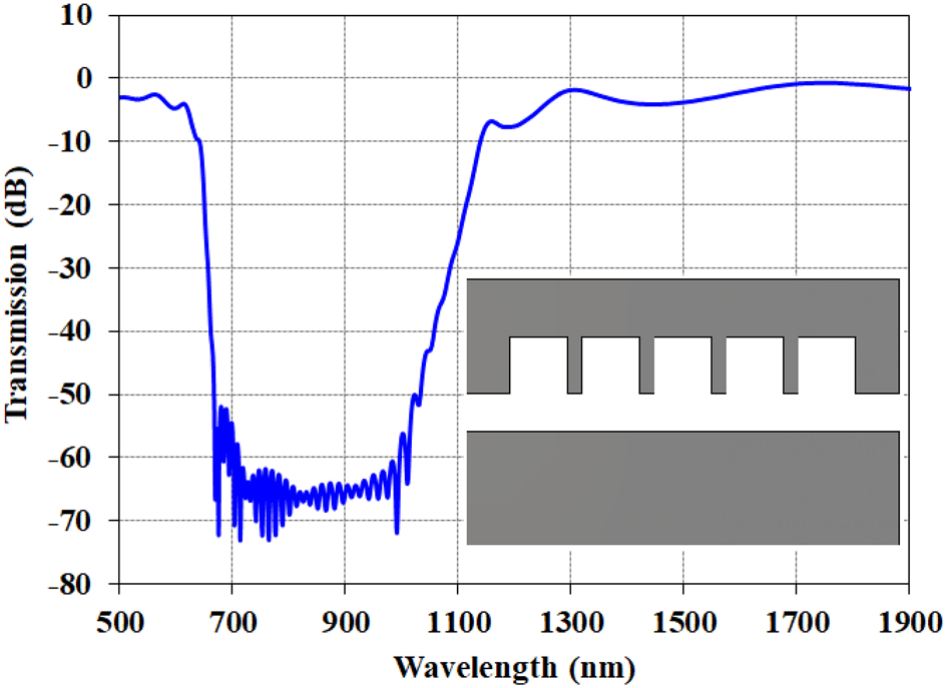
The transmission of the optical waveguide without the H-elements.

In Fig. 9, the structure of the proposed optical waveguide without H-shaped elements is examined. As can be seen here, the transmission response structure of the structure is different from the structure with H-shaped elements and is a wide band filter in the range of 650 to 1130 nm with a minimum transmission of − 72 dB. Therefore, the absence of H-shaped elements changes the dual band structure to single wide band structure also causes an increase in the transmission value, so it is not suitable for sensing applications.

**Figure 10:**
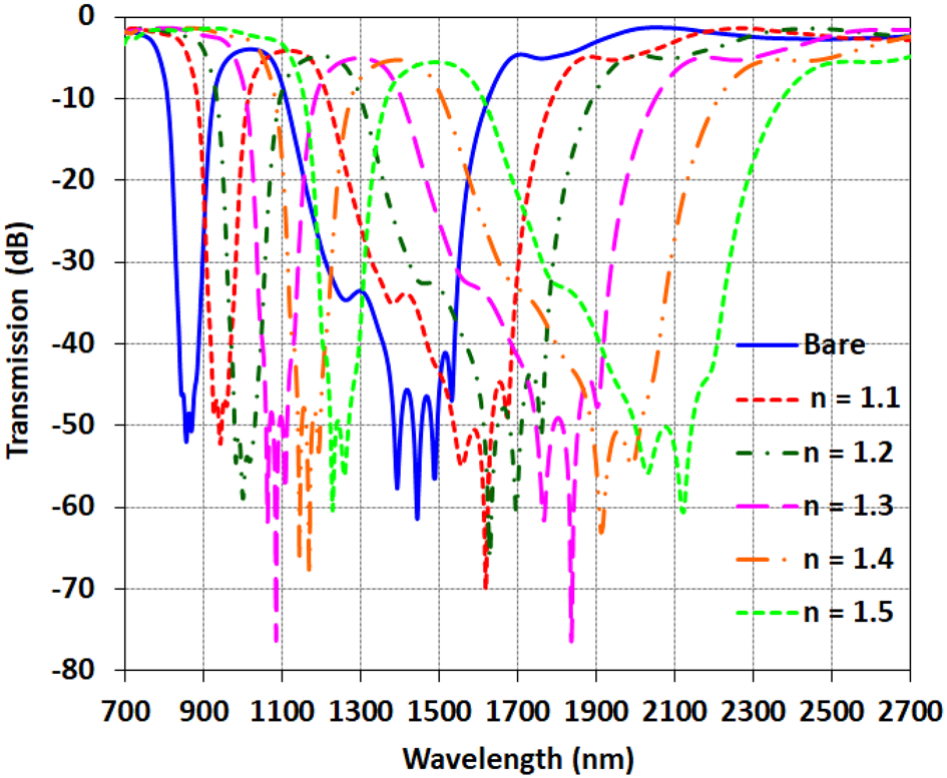
the transmission of the filled waveguide with various refractive index in comparison with the bare sensor.

Figure 10 shows the transmission response of the proposed waveguide structure when empty and filled with the analyte material under test (MUT). It can be observed that as the refractive index increases from 1 to 1.5, the resonance dips exhibit a shift towards longer wavelengths. This redshift in the transmission spectrum as the surrounding refractive index changes forms the basis for the sensing capability. The sensitivity is determined by the magnitude of the wavelength shift per refractive index unit change. The pronounced shifts of the resonant wavelengths in Fig. 8 highlight the potential of the designed coupled-cavity waveguide structure for sensitive refractive index detection. By tracking the displacement of the sharp dual-band resonances, small variations in the refractive index of the analyzed samples can be discerned. Further engineering the cavity and array design can optimize the sensitivity. The unique transmission characteristics arising from the slow-wave effect enable both high sensitivity and filtering functionality. As can be seen in Figs. 8 and 9, many ripples have appeared in the broadband spectrum which is undesirable for sensing applications. Thus two main points are considered for sensing (1) The frequency is chosen as corresponding to the dip for new material. (2) Ripples are so close that the effects of errors can be ignored due to the very high Q of this structure. However, the best way to use this type of structure for sensing is to obtain the average of ripples variation in the broad spectrum.

The performance of a refractive index sensor can be evaluated by two key parameters of the sensitivity and figure of merit (FOM). The sensitivity indicates the shift in the resonance wavelength per change in refractive index (S = Δλ/Δn), with higher values corresponding to more sensitive detection. It is often expressed in nm/RIU, representing the wavelength shift per refractive index unit. The FOM provides an indication of the resonance quality, by accounting the transmission based on FOM = ΔT/T_0_Δn^35,36^. A sharper, narrower resonance enables more accurate tracking of small wavelength shifts, yielding higher FOM values. This parameter reveals the limit of detection—larger FOM values indicate the capability to discern smaller refractive index changes. Table 2 lists the calculated sensitivity and FOM for the two resonances, at wavelengths *f*_1_ and *f*_2_, when the surrounding refractive index is varied from 1.1 to 1.5. The high FOM values, reaching over 20,000, highlight the strong sensing potential based on the sharp resonances of the proposed structure.

**Table 2:** Comparison of the sensitivity and FOM for both resonances corresponding with various refractive indexes.

	*f*_1_		*f*_2_	
	Sensitivity (nm/RIU)	FOM (RIU^−1^)	Sensitivity (nm/RIU)	FOM (RIU^−1^)
n = 1.1	773	2000	1774	20,000
n = 1.2	670	5727	940	5000
n = 1.3	736	10,416	1319	14,580
n = 1.4	697	1071	1176	3076
n = 1.5	727	200	1339	2000

Examining the sensitivity values, it is evident that the *f*_2_ resonance demonstrates higher sensitivity compared to *f*_1_, with values ranging from 940 to 1774 nm/RIU across the analyzed refractive index range. The maximum sensitivity of 1774 nm/RIU is achieved for *f*_2_ at a refractive index of 1.1.

As the index increases, the sensitivity reduces somewhat but remains high, at over 1000 nm/RIU for indices between 1.2 and 1.5. This indicates a robust capability to detect small refractive index changes. The *f*_1_ resonance also provides useful sensitivity, varied from 670 to 773 nm/RIU, demonstrating that both resonances can enable refractive index sensing.

In terms of FOM, extremely high values are attained for both resonances, highlighting the low transmission and high quality factors of the resonances. For *f*_2_, remarkably high FOM on the order of 10,000 to 20,000 RIU^−1^ is obtained. The *f*_1_ resonance also reaches FOM exceeding 1000 RIU^-1^ for several indices. The combined high sensitivity and high Q-factor underscore the potential of the proposed coupled cavity structure for sensitive refractive index detection based on the sharp resonances. Engineering the cavity design could further optimize the sensitivity and FOM.

**Figure 11:**
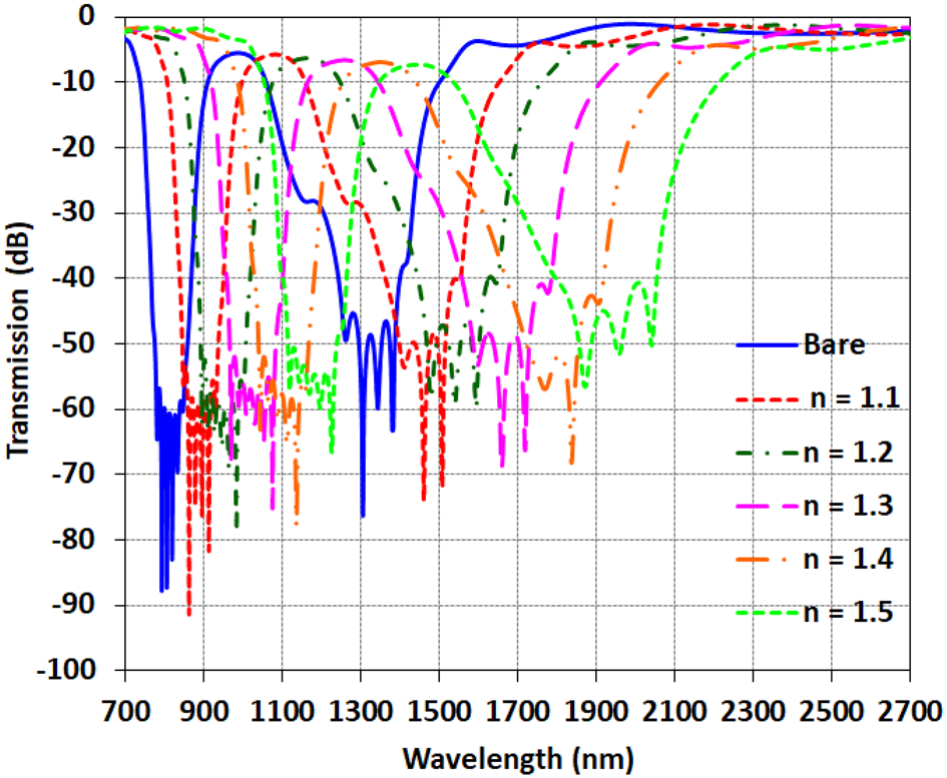
the transmission of the filled waveguide with various refractive index in comparison with the bare sensor for modified model.

The transmission of the filled waveguide with various refractive indexes for n = 1.1 to 1.5 in comparison with the bare sensor for modified model is presented in Fig. 11.

Table 3 shows the calculated sensitivity and figure of merit (FOM) for the two resonances at wavelengths *f*_*1*_ and *f*_*2*_, when the surrounding refractive index is varied from 1.1 to 1.5 for the modified cavity design. Examining the sensitivity values, the *f*_*2*_ resonance again demonstrates higher sensitivity than *f*_*1*_, with values from 1060 to 1324 nm/RIU across the analyzed indices. The maximum sensitivity is 1324 nm/RIU for *f*_2_ at a refractive index of 1.5. As the index increases, the sensitivity reduces somewhat but remains above 1000 nm/RIU from 1.1 to 1.5, indicating maintained high sensitivity for detecting small refractive index changes. The *f*_1_ resonance provides sensitivity ranging from 750 to 806 nm/RIU, slightly improved over the basic design. So both resonances enable useful refractive index sensing.

**Table 3:** Comparisons of the sensitivity and FOM for both resonances corresponding with various refractive indexes.

	*f*_1_		*f*_2_	
	Sensitivity (nm/RIU)	FOM (RIU^−1^)	Sensitivity (nm/RIU)	FOM (RIU^−1^)
n = 1.1	800	115,000	1260	2600
n = 1.2	750	85,000	1060	4000
n = 1.3	806	66,000	1135	5200
n = 1.4	755	49,000	1140	6500
n = 1.5	786	41,000	1324	5400

In terms of FOM, remarkably high values are attained for both *f*_1_ and *f*_2_ resonances. For *f*_2_, the FOM spans 2600 to 6500 RIU^-1^. Although lower than the basic design, these FOM values are still very high. Meanwhile, the optimized *f*_1_ resonance achieves dramatically increased FOM between 41,000 and 115,000 RIU^-1^, owing to the narrowed line-width. The combined high sensitivity and extremely high Q-factors validate the potential of the coupled Fano resonators for sensitive detection of small refractive index changes.

In short, comparison between Tables 2 and 3 shows the advantage of the modified structure for enhancing the FOM of this sensor, however, the basic model shows higher sensitivity. So, the appropriate model can be selected based on the demand for sensitivity or FOM measurement.

This work and other optical waveguides as refractive index sensors are compared in Table 4. The previous studies are considered for comparisons have been done from 2015 to 2023. The type of optical waveguide, developing technique, operation wavelength, and number of resonances, sensitivity, and FOM of these sensors as the main factors that are noticed for this comparison. Cavity structure is known as the conventional type of optical sensor^6,9,10,15,20,37–39^, however, in some studies the stub structures have been developed^2,7,20^. Typically, the cavity is interesting more than the stub model due to their higher sensitivity and FOM. In this study, we have developed a solution for the stub model's drawbacks by combining the slow wave characteristic with the cavity technique in the stubs. So, the FOM is enhanced up to 1.15 × 10^5^ RIU^-1^ while previous studies have been reported between 51 RIU^−1^^7^ up to 9.95 × 10^4^ RIU^−1^^38^ by using the cavity technique with the cross slot. The maximum FOM of 3.51 × 10^4^ RIU^-1^ has been obtained for stub structure by combining with cavity^20^. In this work, the maximum sensitivity of 1774 nm/RIU is achieved and this value is more than the most of studies in this field. However, sensitivity of 6400 nm/RIU^6^ and 2900 nm/RIU^37^ have been reported by implementing the cavity techniques. In short, this comparison and study reveal that the suggested technique can overcome many challenges in developing a sensor in comparison to its ancestor.

**Table 4:** This work comparing with other optical waveguide.

	Type	Technique of design	Wavelength (nm)	Sensitivity (nm/RIU)	FOM (RIU^−1)^
This work	Multi-stub	Slow-wave	850,1450	1774	1.15 × 10^5^
^2^	Dual Stub	Z-shape Stub	1550 ,3150	1791	3800
^6^	Cavity resonator	Coupled line	700	6400	1 × 10^4^
^7^	Dual Stub	Loop form Stub	600,800,1500	1840	51.1
^9^	Cavity	Rectangular ring	800,1100	1300	6838
^10^	Cavity	double-baffle contained	1400	825	5.7 × 10^4^
^15^	Cavity	Dual ring	550,650, 700,900,1300	1290	3.6 × 10^4^
^20^	Stub	Cavity -stub	700,1300	1450	3.51 × 10^4^
^37^	Cavity	Circular ring	475,550,750,860,1080	2900	240
^38^	Cavity	Cross slot	400,600,900,1100	1100	9.95 × 10^4^
^39^	Cavity	T-type and ring	470,1050	1012	5.57 × 10^4^

## Conclusion

This work proposed and analyzed a plasmonic coupled cavity waveguide structure for refractive index sensing applications. The device consists of a MIM waveguide side-coupled to an array of asymmetric split ring resonators designed to induce dual-band resonances. The periodic arrangement of dual-band resonators creates a slow light effect to enhance light-matter interactions within the narrow region between metal layers. The transmission characteristics exhibited two sharp resonant dips associated with the dual-band resonances. As the number of coupled cavities increased from 1 to 5, the resonance depths improved from − 10 dB to over − 60 dB, showcasing the slow light effect. The resonances demonstrated large wavelength shifts as the ambient refractive index was varied, highlighting the sensitivity of the structure. A maximum sensitivity of 1774 nm/RIU and high figure of merit exceeding 2 × 10^4^ RIU ^−1^ were achieved for the basic model and for the modified model, the FOM reached 115,000. Furthermore, engineering the cavity design enabled spectral shaping functionality across a wide bandwidth. The proposed platform can potentially enable both refractive index sensing based on tracking the dual-band resonances, as well as optical filtering on a nanophotonic chip. The simple, compact geometry makes the structure suitable for integration with microfluidics and lab-on-a-chip devices.

## Data availability

The datasets used and/or analyzed during the current study available from the corresponding author on reasonable request.
